# Digitally guided alveolar nerve repositioning surgery: a novel approach to the atrophic posterior mandible

**DOI:** 10.1038/s41415-026-9879-0

**Published:** 2026-09-11

**Authors:** Pynadath George

**Affiliations:** Huddersfield Royal Infirmary, Huddersfield, UK; University of Lancashire, Lancashire, United Kingdom

## Abstract

Inferior alveolar nerve repositioning is an uncommon surgical procedure performed in oral surgery to safeguard the alveolar nerve during certain dental or reconstructive procedures. While this procedure is generally safe and effective, it is important to understand the potential risks and complications associated. This article presents a computer-guided surgical stent to expedite the surgical accuracy while reducing the risks of nerve injury during alveolar nerve repositioning using local anaesthesia alone. In conclusion, the use of a bone supported guide could improve surgical outcomes, although there is an associated additional cost due to its fabrication. More cases need to be reported to determine the full benefit of a guide in relation to complex oral surgery.

## Key points

- Presents a computerised guided approach to nerve repositioning surgery.
- Explains the benefits to using a guide for complex oral surgery.
- Reports a brief explanation on when to undertake nerve repositioning surgery.

## Introduction

Inferior alveolar nerve repositioning (IANR) surgery is a specialised surgical procedure performed in oral surgery to avoid permanent nerve injury during certain dental or reconstructive procedures. The technique involves identifying and protecting the inferior alveolar nerve before repositioning the neurovascular bundle out of its bony canal. The inferior alveolar nerve supplies sensation to the lower lip, chin, and teeth. The procedure was initially presented by Alling in 1977^1^ who described the technique of identifying and excavating the nerve away from the surgical site and protecting it during surgery. The nerve is then replaced back often in a more lateral position. This nerve repositioning technique is commonly described as nerve lateralisation.

The primary benefit of alveolar nerve repositioning surgery is the prevention of nerve injury. By taking proactive measures to reposition the nerve away from the surgical site, this procedure significantly reduces the risk of permanent damage to the nerve. Preserving nerve function leads to improved patient outcomes and increased satisfaction with the surgical results.

Most dental implant surgery procedures will have some level of surgical risks including but not limited to: bleeding, bruising, pain, oedema and the possibility of infection. With IANR surgery there still lies the risk of temporary or permanent damage to the inferior alveolar nerve, leading to dysaesthesia, hypoesthesia, paraesthesia and anaesthesia. This needs to be consented for and stressed to the patient. The presentation of nerve repositioning indicated for dental implants was first presented by Jenson and Nock in 1987.^2^ This technique is different from the technique described by Alling in 1977 as not only is the alveolar nerve repositioned posteriorly and laterally, but it is transected from the anterior incisive branch of the alveolar nerve. This technique is commonly known as nerve transposition. The chances of permanent damage to the nerve is approximately 1.5% for nerve lateralisation and 12% for nerve transposition^3^ with most nerve-related issues resolving at around 6–12 months.^4^

There are several indications for IANR surgery:

- Pre-prosthetic surgery for removable dentures^5^
- Dental implant placement^6^
- Exodontia^7^
- Orthognathic surgery, or other reconstructive procedures involving the lower jaw^8^^,^^9^
- Mandibular resection due to tumours and cysts.^10^

This case report aims to highlight the novel use of a laboratory-made, tooth- and bone-supported, osteotomy guide for transposition of the inferior alveolar nerve. This guide was fabricated using the pre-operative digital imaging and communications in medicine (Dicom) data from a cone beam computed tomography (CBCT) scan and standard tessellation language (STL) file of a digital intra-oral pre-operative scan of the patient's mouth. Computer-aided planning helps to not just locate the inferior alveolar nerve within the mandible but also allows the precise outline of the overlying bone to establish the planned surgical bone cuts. Computer-aided surgical planning also provides a virtual navigation pre-operatively to address and manage possible other difficulties that maybe encountered during the surgery, such as nearby apices of adjacent teeth.^11^

## Patient selection

Patient selection is important in cases of IANR. The patient must be in good psychological health. Patients must also be in good general medical health, and their dental health must be stable. Any medical issues must be well controlled, and any dental pathology should be addressed before any complex oral surgery.

This article focuses on the use of IANR surgery in modern dental implantology. Appropriate justification for IANR are mandibles with such significant bone loss which deem the placement of conventional or even short dental implants impossible. Another justification is that the alveolar nerve is in the direct trajectory of the planned surgical osteotomy.^12^

There are but a few contraindications for IANR. These include:

- Limited access to the surgical site (for example, mouth opening)
- Thin or very lingually-placed inferior alveolar nerve
- The quantity of bone is so reduced where there is a high possibility that providing IANR surgery could lead to iatrogenic fracture of the mandible.^13^

The alternative to IANR in mandibles with significant bone loss is bone grafting. However, grafting can fail or resorb and is also associated with high rates of complications with reports of over 25%.^14^ Even in successful outcomes of bone grafting there are often longer treatment times and an increased number of appointments, before achieving a dentition supported with implants. IANR offers a cost-effective and quicker approach in comparison albeit with higher risks to the alveolar nerve. This can be highly relevant for those patients wanting quicker treatment times.

## Case report

### Pre-operative assessment and planning

**Fig. 1:**
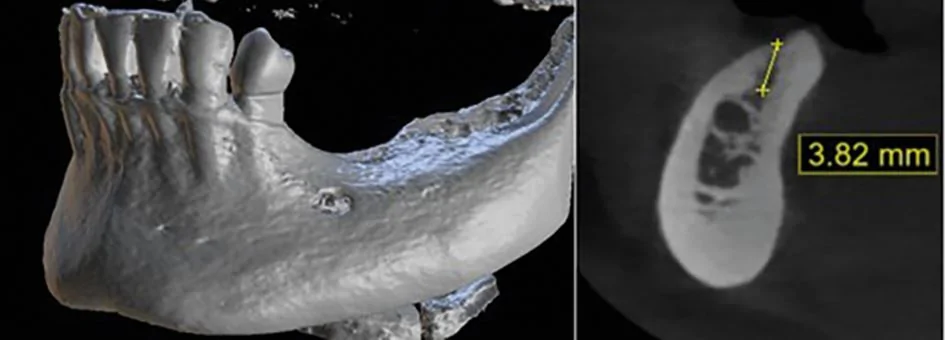
Cone beam computed tomography render of the pre-operative situation. Mental foramen just posterior to the lower left first premolar

A fit and well, 40-year-old woman with no known drug allergies, requested implant-supported teeth to replace the missing dentition in the lower left posterior mandible. These teeth had been lost due to caries several years previously leaving severe bony atrophy to the existing alveolus. There was less than 4 mm of bone superior to the alveolar nerve and canal. This was unsuitable for both conventional and short implants to be installed (Fig. 1). The patient had declined a removable denture and did not wish to undergo multiple appointments that would have been associated with vertical and horizontal bone grafts. As such the patient opted for IANR and the associated risks involved.

**Fig. 2:**
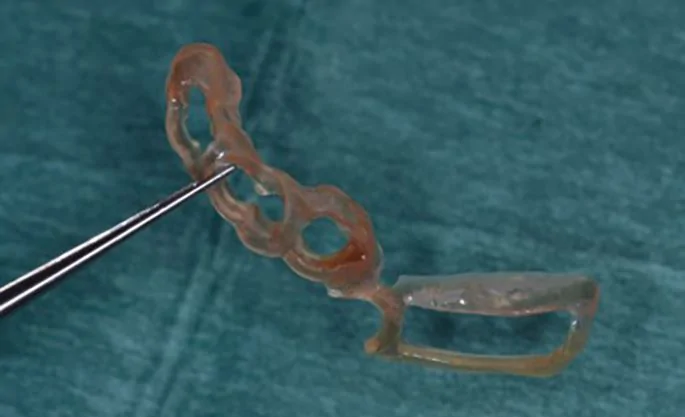
Cone beam computed tomography and standard tessellation language-guided surgical stent

After merging of the DICOM and STL files of the patient, a surgical guide was fabricated outlining the inferior alveolar nerve path within the mandible. A window outline was marked in the guide to highlight the inferior nerve canal and the mental foramen. The guide was designed to be tooth and bone supported to increase its stabilisation during surgery (Fig. 2).

### Surgical technique

**Fig. 3:**
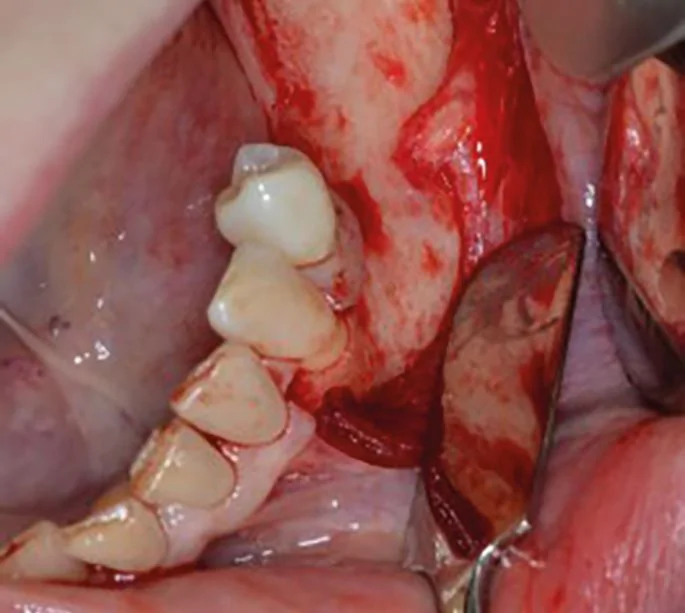
Exposure of mental nerve
- Once adequate surgical access was gained to the mandibular bone the surgical guide was seated and assessed for stability (Fig. 4). The window in the surgical guide to identify the inferior alveolar canal was marked on the bone cut with a piezotome unit

- The procedure commenced with an inferior alveolar block injection using 2.2 ml lignocaine 1:80,000 adrenaline. A further 4.4 ml of the same lignocaine was infiltrated both buccal and lingual to the lower left canine to second molar region. 4.4 ml of articaine 1:100,000 was infiltrated further in the buccal keratinised tissues
- A careful crestal incision was made in the edentulous space distal to the 34 to 38 region with an anterior relief to 33 preserving the adjacent papillary base
- A full thickness flap was raised identifying the mental foramen (Fig. 3)

**Fig. 4:**
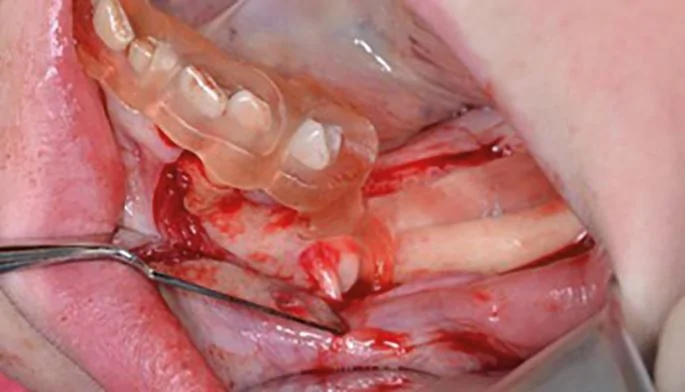
Fitting of the surgical guide
- Two bony windows were made; one for the mental foramen and one for the bone overlying the inferior alveolar nerve canal (Fig. 5)

**Fig. 5:**
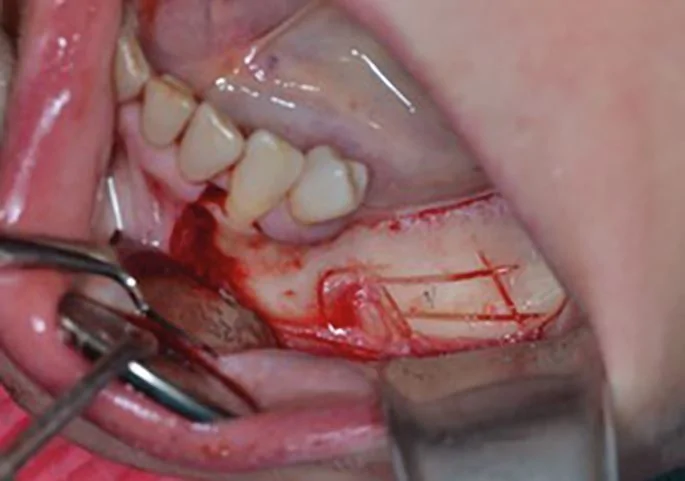
Piezoelectric cuts into the bone
- Once both bony windows were removed, the full extent of the alveolar nerve could be visualised and mobilised using latex and Lucas curettes (Fig. 6)

**Fig. 6:**
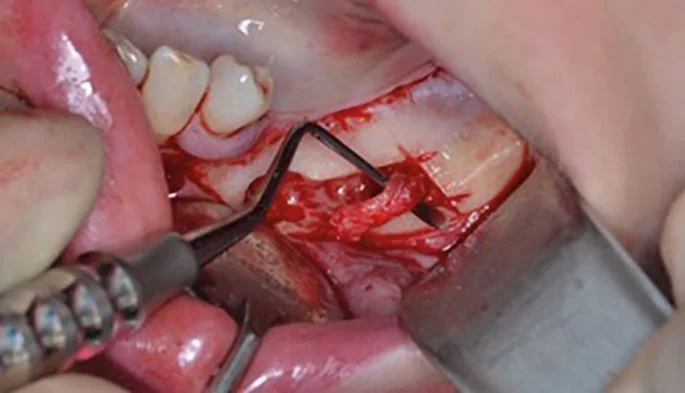
Mobilisation of inferior alveolar nerve
- The alveolar nerve was the transected from the anterior incisive branch before transpositioning it posteriorly
- The existing alveolar nerve canal within the mandible was the grafted with an allograft and an overlying collagen membrane before the flap was closed with simple interrupted sutures using 4.0 vicryl (Fig. 7).

**Fig. 7:**
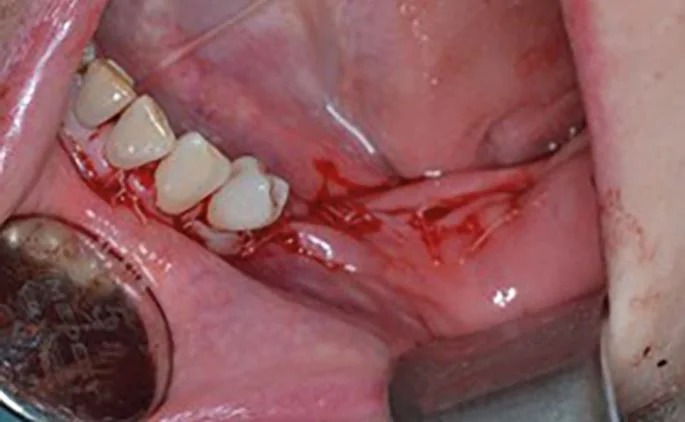
Primary closure

### Post-operative care and management

**Fig. 8:**
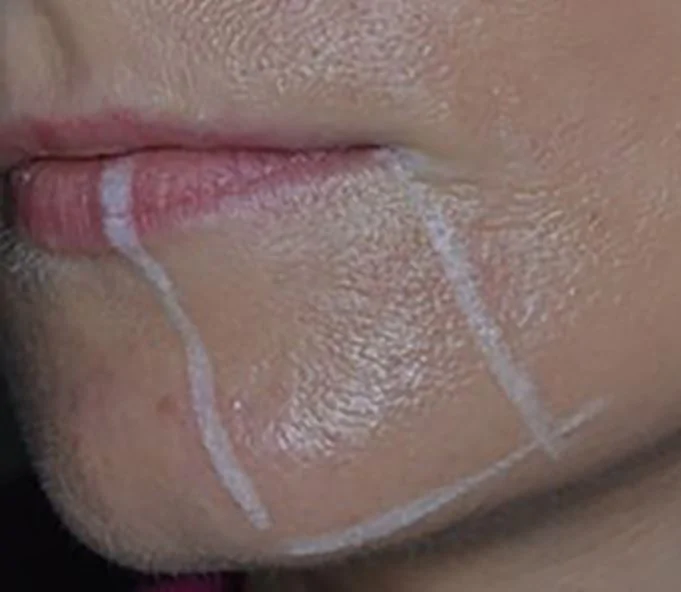
The initial area of hypoaesthesia before full resolution

**Fig. 9:**
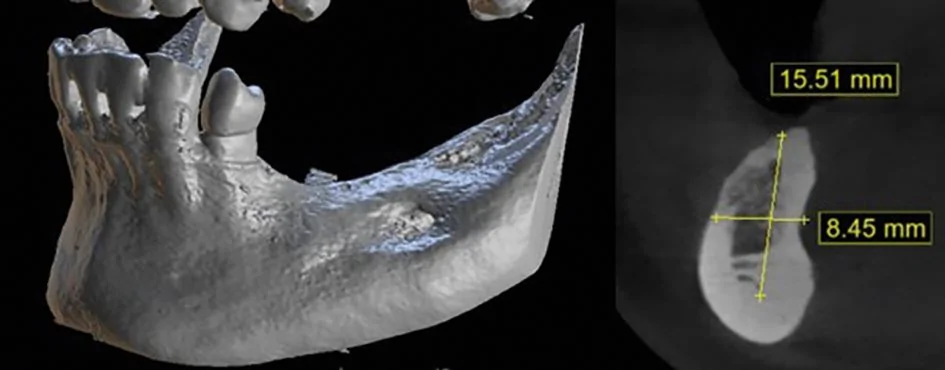
Cone beam computed tomography render of the post-operative situation. The ‘new' mental foramen now posterior from the original mental foramen. The buccal bone has completely integrated and healed

The patient was provided with detailed post-operative instructions. These included managing potential bruising and swelling, dietary recommendations. Prescriptions for amoxicillin 500 mg TDS for seven days, and 1 g of paracetamol QDS for seven days were provided, along with 6 mg of dexamethasone daily for three days. After two weeks the patient was reviewed and healing was assessed. The patient continued to be seen for neurosensory tests involving two-point discrimination tests, which were mapped and recorded every three months to assess nerve responses (Fig. 8). All nerve-related issues had resolved at nine months, and a CBCT scan was taken to confirm full bony healing within the mandible before the installation of dental implants (Fig. 9).

## Discussion

This paper describes a novel approach to IANR surgery, using a surgical guide designed from a CBCT Dicom file and a STL file of an intra-oral scan. Due to the accuracy of this guide, the procedure can be undertaken in almost half the time it would usually take for the author to complete. This efficiency in surgical time also provides confidence to complete the procedure under simple local anaesthetic alone, contradicting previous recommendations of general anaesthesia.^15^ The steps illustrated in this article highlight the evolving technology and techniques for this unique surgical procedure.

Of course, there can be drawbacks to the use of a guide for surgery. Guides increase the costs of surgery. With modern printing techniques rather than subtractive fabrication, the costs of a guide can be minimal and easy to produce. Other drawbacks include the fact that guides can be inaccurate. This is because if the guide fits onto soft tissue rather than hard tissue, such as tooth or bone, it can result in movement of the guide, leading to inaccuracy.^16^

IANR presents a unique alternative to significant bone grafting for the posterior mandible especially for the severely resorbed mandible.^17^ In 2025 a new classification for nerve repositioning for dental implants was proposed by Kablan.^18^ According to this classification, there are four categories of the mandible based on the quantity of bone available; this single case report is category one. The classification also provides a treatment protocol for each type of mandible. The limiting aspect of this classification is it does not account for the position of the nerve within the mandible or the quantity of bone below the alveolar nerve. These two factors are of the utmost importance for several reasons. A nerve which is lingual within the mandible will require more nerve mobilisation than a nerve which is buccal. This increases the risks of nerve trauma. In addition, a nerve with only 3 mm of residual bone inferior to it, compared to 6 mm, risks the mandible's integrity before fracture.

The decision to undertake IANR was based on several factors, including the desire to reduce surgery time. The main disadvantage to IANR is nerve-related trauma. This trauma can occur due to improper surgical access to the alveolar nerve if the osteotomy is incorrect. This can be minimised by a guide improving surgical accuracy.^19^

Nerve damage is associated with the type of nerve repositioning surgery. Nerve lateralisation has been associated with just 3% of long-term neurosensory deficiencies compared to 22% which was associated with nerve transposition.^20^ Rotary instrumentation results in higher chances of permanent nerve-related signs and symptoms and should be avoided if possible and substituted with piezotomes. This will help avoid nerve damage through mechanical and thermal trauma often associated with rotary instruments while the overlying bone to the nerve is being removed.^21^

Post-operative care is important as infection and mandible fracture can occur. As such it is recommended to follow-up closely with further examination and radiological scans of the surgical site if there are signs and symptoms of poor healing or fracture. With guided stents the removal of bone to access the alveolar nerve is minimised^22^ and therefore reducing the risks of mandibular fracture intra- and post-operatively.

## Conclusion

Providing nerve repositioning surgery requires a significant amount of skill and training due to the challenging nature of the surgery. Technology and equipment have evolved over time to improve the accuracy, efficiency and success rates of such a valuable technique but this is not a substitute for the competencies required. Surgeons should stay up to date with current concepts and education if they are eager to gain this skill set. Finally, this article contributes to the changing evidence base to nerve repositioning surgery, offering insights on how to provide a relatively complex surgery more effectively and establishing new approaches.
